# Establishing consensus on principles and competencies for the use of play in clinical practice in hospitals: An international Delphi study

**DOI:** 10.1007/s00431-023-05411-4

**Published:** 2024-01-06

**Authors:** Jakob Thestrup, Jette Led Sørensen, Jane Hybschmann, Martha Krogh Topperzer, Kelsey Graber, Christine O’Farrelly, Jenny Gibson, Paul Ramchandani, Thomas Leth Frandsen, Line Klingen Gjærde

**Affiliations:** 1 Mary Elizabeth’s Hospital and Juliane Marie Centre, Copenhagen University Hospital – Rigshospitalet, Juliane Maries Vej 4, 2100 Copenhagen, Denmark; 2 Department of Clinical Medicine, Faculty of Health and Medical Sciences, University of Copenhagen, Copenhagen, Denmark; 3 Educational Unit, Copenhagen University Hospital – Rigshospitalet, Copenhagen, Denmark; 4 Centre for Research on Play in Education, Development & Learning (PEDAL), Faculty of Education, Cambridge University, Cambridge, UK

**Keywords:** Play, Healthcare professionals, Children, Adolescents, Paediatrics consensus, Delphi

## Abstract

Although play has existed in paediatric hospitals for decades, a shared understanding of why and how healthcare professionals use play in clinical practice is lacking. This study aims to reach consensus on a common set of principles and competencies for play interventions and practices in hospitals. We conducted a three-round Delphi study that included healthcare professionals selected by hospital management. The first round comprised open-ended questions on the use of play in clinical practice. Principles and competencies, including learning objectives, were established using content analysis through an iterative process. Participants rated the importance of each principle and learning objective in the second and third rounds. Among the 66 participants, 45 (68%) responded in round 1 and 41 (62%) in rounds 2 and 3. The participants represented ten countries and nine different health professions. After the three rounds, we identified 33 principles and six overall competencies: building trusting relationships; delivering information and increasing understanding; promoting cooperation and participation; reducing procedure-related anxiety and pain; supporting coping and development; and ensuring a professional approach to play, which comprised 20 learning objectives.

*  Conclusion*: According to healthcare professionals, play in clinical practice can be used to communicate and build relationships with paediatric patients and thus potentially help provide patient-centred care. Our findings may help guide and prioritize future research initiatives and operationalize play interventions and practices in hospitals.

**Table:** 

**What is Known:** *• Evidence suggests that using play in clinical practice can help paediatric patients during hospitals stays.* *• Despite the evidence supporting the use of play, a shared understanding of why and how paediatric healthcare professionals use play is needed.*	
**What is New:** *• This international Delphi study contributes to a shared interprofessional understanding of the principles, competencies and learning objectives for the use of play in clinical practice.* *• The findings have the potential to aid initiatives in developing training programmes for healthcare professionals in using play to provide care with a patient-centred approach.*

**Supplementary Information:**

The online version contains supplementary material available at 10.1007/s00431-023-05411-4.

## Introduction 

Play is increasingly seen as a valuable approach to help children and adolescents cope with the challenges of hospitalization [1]. The European Association for Children in Hospital includes play among the ten principles to promote children’s rights in hospitals [2]. Moreover, the World Health Organization (WHO) recommends that all doctors and nurses regularly use play in treatment and care [3]. In a previous review of 297 studies, we identified four clinical contexts where play interventions had a specific role in hospitals: procedures and diagnostic tests, patient education, treatment and recovery and adaptation (diversional or coping activities) [4].

A wide variety of healthcare professionals conduct play interventions. Some countries have play or child life specialists who use play in the clinic but typically only in specific situations (e.g. preparing paediatric patients for procedures like injections) or hours of the day. Thus, doctors, nurses, physiotherapists and other staff often use play in clinical practice [4] but generally without formal training. This lack of training is a barrier to using play effectively [5] and suggests a need to equip paediatric healthcare professionals with the competencies to integrate play into clinical practice.

A recent review highlighted a general lack of structured approaches for integrating play into healthcare education, with only few educational programmes available [6]. Moreover, a knowledge gap exists regarding how and why healthcare professionals integrate play into clinical practice [1, 4]. This amplifies the need for a shared interprofessional understanding of the use of play in hospitals to guide educational initiatives and operationalize local play interventions and practices. Therefore, this Delphi study aims to reach consensus among healthcare professionals on the principles and competencies for play interventions and practices in hospitals internationally.

## Materials and methods

### Study design

Based on the Standards for Quality Improvement Reporting Excellence [7], this three-round Delphi study was conducted to establish consensus regarding the principles and competencies for using play in hospitals with an international panel of healthcare professionals selected by management. The Delphi method is a well-known and well-accepted method used to achieve consensus on topics that lack an established knowledge base [8]. Medical education research commonly uses consensus methods to identify curricular content, e.g. competencies and learning objectives [9, 10]. An advantage of this technique is that participant anonymity prevents participants’ dominance by giving everyone an equal opportunity to express their opinion [11]. Adherence to Humphrey-Murto et al.’s 11 recommendations ensured methodological rigor [12]. The study protocol and 11 recommendations are available on the Open Science Framework [13].

### Selection of expert panel

We used purposive sampling to recruit healthcare professionals from paediatric departments and hospitals in Australia, Canada, the USA and eight European countries (Denmark, Germany, Ireland, Italy, the Netherlands, Norway, Spain and the UK). Target hospitals were chosen from *Newsweek’s* World’s Best Specialized Hospitals–Pediatrics (2021) [14] and through the European Children’s Hospitals Organisation [15]. Hospital leaders, heads of clinical services and head nurses were invited to appoint 3–10 Delphi participants with expertise and/or experience in using play, aiming to recruit 60–80 participants. To ensure broad professional representation, we encouraged the appointment of professionals with various clinical backgrounds, all of whom received a study invitation in their native language.

### Data collection

The study comprised three rounds of electronic surveys using Research Electronic Data Capture tools [16] from March to September 2022. We invited all participants to participate in each round. The first-round questionnaire was professionally translated using forward–backward translation [17] and distributed in eight languages (Danish, Dutch, English, French, German, Italian, Norwegian and Spanish), allowing participants to answer in their native language. A professional translator did the initial forward translation from English to each target language. For quality control, another professional translator then back translated into English. Finally, two independent reviewers compared the translations for accuracy and consistency jointly with bilingual healthcare professionals. Rounds 2 and 3 were in English, but participants could request a version in their native language. Each round contained demographic questions on age, country of origin and clinical background. In all rounds, participants were welcome to provide open-ended feedback on the wording or inclusion of missing statements related to the use of play in hospitals. To increase the response rate, weekly reminders were sent during each round.

**Fig. 1:**
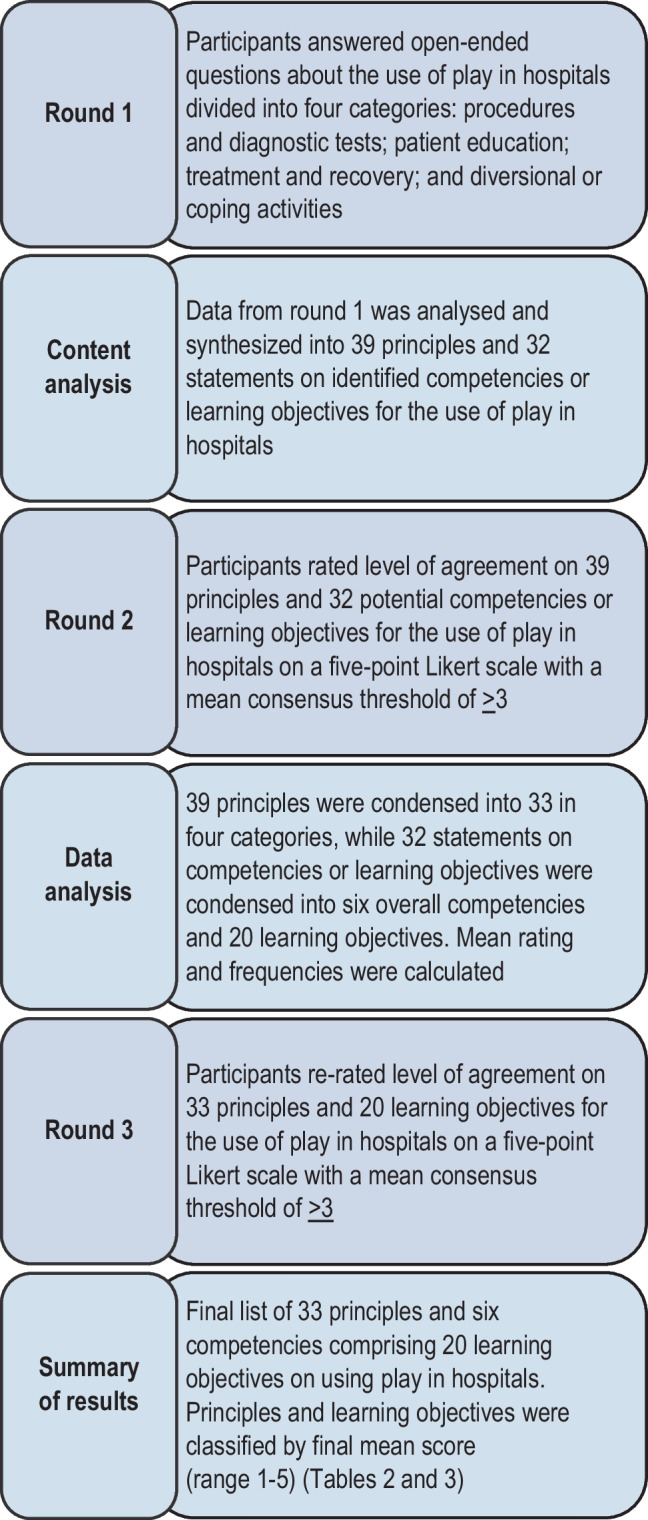
Overview of the steps included in the Delphi process

In *round 1*, participants were asked open-ended questions about their use of play that were divided into four clinical contexts: procedures and diagnostic tests, patient education, treatment and recovery and adaptation [4]. We asked about factors considered before using play and about facilitators and barriers to integrating play. Supplementary Table 1 and Fig. 1 show the questionnaire.

Between rounds 1 and 2, the qualitative data in Dutch, German, Italian and Spanish were translated into English using Google Translate (English, Danish and Norwegian are native languages in the research team). When words or sentences were unclear, we used a dictionary to determine the appropriate definition and discussed any issues among the authors conducting the analysis and bilingual healthcare professionals. No participants answered in French. All data were then analysed and synthesized into statements about play in hospitals. Round 2 asked participants to rate each statement on a five-point Likert scale: 1 = not relevant, 2 = less relevant, 3 = relevant, 4 = very relevant, and 5 = extremely relevant. Consensus for inclusion was defined as items with a mean rating ≥ 3. Participants were also asked to select the eight most important items on each separate list of factors, barriers and facilitators identified in round 1.

*Round 3* included round 2 statements agreed upon by consensus (≥ 3). Each statement was presented with the mean rating, and participants were asked to rerate relevance using the same 5-point Likert scale as in round 2. Finally, participants were asked to reselect the eight most important factors, facilitators and barriers from a ranked list.

### Data analysis

An analysis was conducted between each round. Three authors (JT, JLS, LKG) independently read the responses to open-ended questions and used content analysis to identify statements without the use of preconceived categories [18]. Three authors (JT, JLS, LKG) analysed the statements collaboratively through iterative discussions with the interprofessional and international author group. We organized the statements into principles for play in hospitals using an iterative process, grouping them into categories emerging from the data analysis. We also identified competencies and learning objectives. We defined competencies in accordance with Lingard et al. [19] such that competence is a context-free quality that individuals acquire and possess. We then used the definition to formulate learning objectives using the six-step framework for writing objectives [20], incorporating an action verb to make them clear and measurable. The learning objectives describe observable actions that healthcare professionals perform after an educational activity.

Descriptive statistics were performed in RStudio, version 1.4 [21], and mean scores and standard deviations were obtained for all statements. We calculated the frequency of occurrence for the factors, facilitators and barriers identified. The mean ratings and frequencies were reported to the participants in round 3.

## Results

### Participant characteristics

Sixty-six healthcare professionals were invited to participate in the Delphi study, 45 (68%) of whom completed round 1, while 41 (62%) completed rounds 2 and 3. The participants, representing nine health professions, were from Australia, Canada and eight European countries. Table 1 presents the demographics of the participants in each round.

**Table 1:** Participant characteristics in each Delphi round

Characteristic	Round 1 *n* (%)	Round 2 *n* (%)	Round 3 *n* (%)
**Number of participants**	45 (68)	41 (62)	41 (62)
**Age**			
20–29	2 (4)	2 (5)	2 (5)
30–39	8 (18)	9 (22)	8 (19)
40–49	14 (31)	14 (34)	15 (37)
50–59	16 (36)	12 (29)	13 (32)
60–69	5 (11)	4 (10)	3 (7)
**Sex**			
Female	43 (96)	39 (95)	39 (95)
**Country**			
Australia^a^	3 (7)	3 (7)	3 (7)
Canada^b^	6 (13)	5 (12)	4 (10)
Denmark^c^	12 (27)	11 (27)	12 (29)
Germany^d^	5 (11)	4 (10)	4 (10)
Ireland^e^	5 (11)	5 (12)	5 (12)
Italy^f^	4 (9)	3 (7)	3 (7)
Norway^g^	2 (4)	2 (5)	2 (5)
Netherlands^h^	3 (7)	2 (5)	2 (5)
Spain^i^	5 (11)	3 (7)	3 (7)
UK^j^	0 (0)	3 (7)	3 (7)
**Health profession**			
Child life specialist/play specialist	8 (18)	8 (20)	6 (15)
Dietician	1 (2)	1 (2)	1 (2)
Doctor	9 (20)	9 (22)	9 (22)
Nurse	7 (15)	5 (12)	7 (17)
Occupational therapist	5 (11)	5 (12)	5 (12)
Clinical social worker	5 (11)	4 (10)	4 (10)
Physiotherapist	3 (7)	3 (7)	3 (7)
Psychologist	6 (16)	5 (12)	6 (15)
Teacher	1 (2)	1 (2)	0 (0)

^a^The Children’s Hospital at Westmead

^b^Children’s Hospital of Eastern Ontario

^c^Copenhagen University Hospital – Rigshospitalet

^d^Dr. von Hauner Children’s Hospital

^e^Children’s Health Ireland at Temple Street and Children's Health Ireland at Crumlin

^f^Meyer Children’s Hospital

^g^Oslo University Hospital

^h^Erasmus MC Sophia Children’s Hospital

^i^Sant Joan de Déu Barcelona Children’s Hospital

^j^Great Ormond Street Hospital

### Consensus statements

#### Round 1

Following content analysis of responses to open-ended questions from round 1, we synthesized 71 statements about using play in hospitals, dividing them into 39 principles about play in hospitals and 32 statements on competencies or learning objectives for using play.

#### Round 2

In round 2, it was agreed to include all principles and statements regarding competencies and learning objectives. After analysing and reorganizing the principles from 39 to 33, we divided them into four overall categories based on context and the Delphi participants’ feedback: children, adolescents, healthcare professionals and organization (Table 2). We reorganized the 32 statements on competencies and learning objectives into six overall competencies comprising 20 learning objectives.

**Table 2:** Principles for play in hospitals divided into four main categories

**Categories**	**Principles for play in hospitals**	**Mean rating**
Children	Play is a way of creating a sense of normalcy for paediatric patients	4.8
	Play is a way for paediatric patients to cope with their hospital experience	4.8
	Play supports normal development in paediatric patients while in hospital	4.8
	Free play is important for paediatric patients	4.7
	Play is useful for paediatric patients during long waiting periods in the hospital	4.7
	Play contributes to social well-being for paediatric patients	4.7
	Play can promote interactions with other paediatric patients	4.6
	Play promotes continuity with everyday life for paediatric patients	4.5
	Play allows paediatric patients to experience ‘childhood’ despite being hospitalized	4.5
	Playful activities or toys from home promotes a comfortable hospital environment for paediatric patients	4.5
	Play can promote interactions between paediatric patients and their siblings	4.4
Adolescents	It is necessary for healthcare professionals to promote play and activities for adolescents	4.1
	The use of play and activities for adolescent patients is often overlooked	4.0
	The use of play and activities for adolescent patients is lacking	3.8
Healthcare professionals	Play can be a way of communicating with paediatric patients	4.6
	Play is a motivator for getting hospitalized children out of bed	4.6
	Play is a way for healthcare professionals to establish relationships with paediatric patients	4.5
	Play allows the expression of non-verbalized emotions and experiences for paediatric patients	4.5
	Play is a way for healthcare professionals to build trust with paediatric patients	4.5
	Play can be used as a tool to evaluate paediatric patients’ physical and mental state	4.5
	Play is a way to help paediatric patients express their emotions	4.4
	Play is a tool for increasing understanding among paediatric patients	4.3
	Play interventions should be applied in close collaboration with the families of paediatric patients	4.3
	Play can be used to reduce anxiety among family members	4.3
	Play offers opportunity for family participation in paediatric patients’ treatment and care	4.2
	Play used to deliver information about an illness makes it easier for paediatric patients to understand their illness	4.1
	Interprofessional communication and collaboration is crucial for the use of play in paediatrics	4.1
	Play is inappropriate if patients clearly demonstrate that they don’t want to play	3.8
	Play is inappropriate in severe clinical emergencies	3.4
Organization	All healthcare professionals should have access to appropriate toys and activities to integrate play in paediatrics	4.3
	All healthcare professionals should have access to formal education on integrating play in paediatrics	4.2
	Educational programmes about integrating play should be customized to individual healthcare professional groups	4.0
	Only some groups of healthcare professionals should have access to formal education on integrating play in paediatrics^a^	3.7

Consensus for inclusion was defined as principles with a mean rating ≥ 3

^a^Item phrased in reverse. To get the reversed scored value, we added one to the maximum possible score and then subtracted the original score of 2.3

#### Round 3

In round 3, all 33 principles and six overall competencies comprising 20 learning objectives were accepted by consensus among the healthcare professionals for the final list. Aside from minor wording changes, no significant new feedback was provided. Figure 1 shows the steps of the Delphi process, and Supplementary Fig. 2 presents the details.

### Principles about play in hospitals

The 33 principles were divided into the four main categories presented in Table 2, also indicating the final mean scores for the principles.

### Healthcare professionals’ competencies and learning objectives for play in hospitals

The healthcare professionals agreed on 20 learning objectives, which we divided into the six main competencies shown in Table 3, also indicating the final mean scores for the learning objectives.

**Table 3:** Healthcare professionals’ learning objectives for play in hospitals divided into six main competencies

**Competencies**	**Learning objectives for play in hospitals**	**Mean rating**
1. Building trusting relationships	Healthcare professionals should be able to use play to reduce anxiety and distress in paediatric patients	4.5
	Healthcare professionals should be able to use play to build trusting relationships with paediatric patients	4.5
2. Delivering information and increasing understanding	Healthcare professionals should be able to use play to prepare paediatric patients for different clinical situations	4.5
	Healthcare professionals should be able to use play to communicate with paediatric patients of different ages	4.4
	Healthcare professionals should be able to use play to educate paediatric patients and their parents about the patient’s disease and strategies to manage treatment and care	4.3
	Healthcare professionals should be able to use play as a way to deliver information to paediatric patients	4.2
3. Promoting participation and cooperation	Healthcare professionals should be able to communicate with the families of paediatric patients to integrate play in their child’s care	4.4
	Healthcare professional should be able to use play to motivate paediatric patients to participate in activities	4.2
	Healthcare professionals should be able to motivate parents to accept the use of play in paediatrics	4.1
4. Reducing procedure-related anxiety and pain	Healthcare professionals should be able to use play as distraction to help paediatric patients during different clinical situations	4.5
	Healthcare professionals should be able to use play as a non-pharmacological method to reduce the need for sedation in paediatric patients	4.5
	Healthcare professionals should be able to use play to reduce pain in paediatric patients	4.5
5. Supporting coping and development	Healthcare professionals should be able to use play to promote paediatric patients’ ability to cope with their hospitalization	4.5
	Healthcare professionals should be able to use play to promote a sense of normalcy for paediatric patients	4.4
	Healthcare professionals should be able to use play as a tool to give paediatric patients a sense of control in a clinical setting	4.4
	Healthcare professionals should be able to use play to explore emotions and feelings of paediatric patients	4.3
	Healthcare professionals should be able to use play to support the development in paediatric patients	4.3
	Healthcare professionals should be able to use play to establish activities during long waiting periods for paediatric patients	4.2
6. Ensuring a professional approach to play	Healthcare professionals should be able to collaborate across professions to integrate play in paediatrics	4.5
	Healthcare professionals should ask the paediatric patient for consent before introducing play	3.6

Consensus for inclusion was defined as learning objectives with a mean rating ≥ 3

### Factors to consider before using play in hospitals

**Fig. 2:**
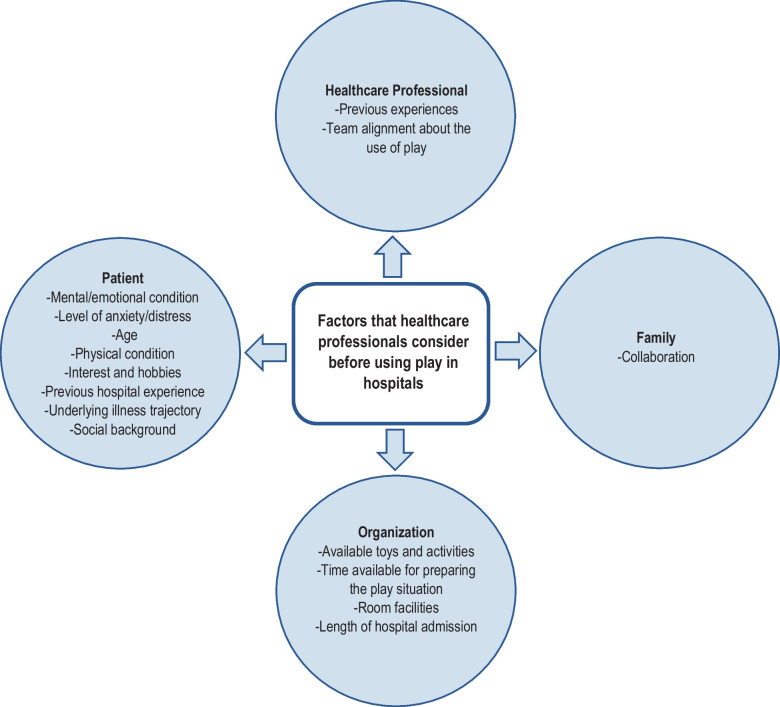
Factors that healthcare professionals should consider before using play in hospitals identified through the Delphi process and divided into four main categories

The participants identified 15 factors that healthcare professionals should consider before using play, the most important of which were patient-centred, including mental and physical state, anxiety level, age and preferences (interests and hobbies). Figure 2 shows the division of factors into four main categories, and Supplementary Table 2 provides a ranked list of the factors, including frequencies.

### Facilitators and barriers to the use of play in hospitals

**Fig. 3:**
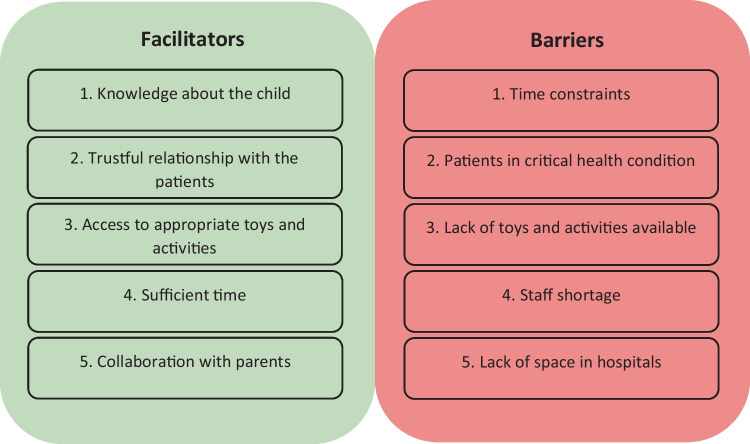
The five most important facilitators and barriers identified through the Delphi process for the use of play in hospitals

The participants identified 16 barriers and 23 facilitators regarding the use of play. The most important barriers were associated with limited organizational resources or critical health conditions. The most important facilitators were related to knowledge about patients and having a trusting relationship, adequate organizational resources and parental collaboration. Figure 3 presents the five most important facilitators and barriers to the use of play in hospitals, and Supplementary Tables 3 and 4 provide complete lists with frequencies.

## Discussion

A three-round Delphi technique enabled an international group of healthcare professionals to reach consensus regarding principles, competencies and learning objectives for using play in hospitals. Healthcare professionals with nine different backgrounds participated, revealing that play was similarly employed and valued across professions and nationalities. There was consensus among the participants that play helps healthcare professionals communicate with paediatric patients, although playful activities for adolescents are often overlooked. The participants agreed that play can be used to build trusting relationships, deliver information and increase understanding, promote cooperation and participation, reduce procedure-related anxiety and pain and support coping and development. Knowledge about patient background and preferences was deemed the most important facilitator for integrating play in clinical practice, while the most important barrier was time constraints.

Many of the identified principles about play in hospitals are closely linked to patient-centred care, which generally advocates planning healthcare around the patient and acknowledging the patients’ right to be fully involved in treatment and care [22, 23]. According to the WHO, developing good communication skills and the ability to form partnerships with patients are essential competencies for providing patient-centred care [24]. However, communication with paediatric patients is particularly complex due to large developmental variations, combined with triadic relationships with parents [25]. In our study, the participants agreed that play was used intuitively to facilitate age-appropriate communication, simplify medical terminology to make information more understandable for paediatric patients and parents, educate about disease management and prepare for various clinical situations. The use of play is well-established to prepare paediatric patients for clinical procedures (e.g. venepuncture or MRI scans) in some paediatric hospitals, along with distraction, which can reduce anxiety and feelings of pain [26–28].

The identified learning objectives align with Gerteis et al.’s [23] seven core elements of patient-centred care, which the Picker Institute also adopted [29]. They include healthcare professionals’ ability to give patients the information they need to understand their illness and treatment, establishing trusting relationships, involving patients and their families in decisions and providing a supportive environment through physical and emotional comfort. Our findings indicate that playful approaches can be an integral part of clinical staff training on patient-centred healthcare in paediatric environments.

Our finding that play promotes paediatric patients’ participation in their own treatment and care also strongly aligns with the promotion of children’s rights in hospitals. The right to participation is a fundamental principle in the UN Convention on the Rights of the Child [30]. Healthcare professionals can promote this right by bringing patients into conversations and decisions in a child friendly manner [31–33]. The recently published rights-based standards for paediatric tests, treatments, examinations and interventions also emphasize this, asserting that healthcare professionals should always communicate directly and ensure that paediatric patients understand, supporting their involvement in choices and acting accordingly [34]. By engaging in play activities, healthcare professionals signal to patients that they are approachable and willing to understand their perspectives and relate on a personal level. This provides a safe space for paediatric patients to express themselves and thereby increase participation in their own care. The participants also agreed that play interventions offer opportunity for increased family participation and noted that play should ideally be conducted in close collaboration with families to ensure engagement and mutually beneficial relationships. Partnerships with family members are also often emphasized in paediatric patient-centred care [35, 36].

Despite the potential benefits of using play in paediatric care, the participants agreed that activities for adolescents are often lacking and overlooked. Interruptions to school life and friendships can have a real impact on the hospitalized youths’ mental health [37, 38], necessitating patient-centred interventions to help them cope. Other studies have found that age-appropriate activities like music are commonly used by adolescents to cope with hospitalization [39], and music therapy supports adolescents with mental disorders and potentially reduces depressive symptoms [40, 41]. However, more research is needed involving adolescents to provide insights into meaningful opportunities for play and recreation in hospital settings for this age group.

For healthcare professionals to integrate play into clinical practice, we found that the most important factors to consider are the patient’s mental and physical state, age, interests and hobbies. The participants also cited knowledge about the patient (interests, hobbies, social background) as the most essential facilitator for integrating play. This suggests a need to incorporate such knowledge in patient records. The most important barriers to using play are generally related to organizational resources, including insufficient time, lack of toys and activities and staff shortages. Time pressure has previously been found to be the most important barrier for the use of play, as reported by nurses [42].

Another identified barrier was healthcare professionals’ lack of education and formal training. A previous study also found that a lack of training made healthcare professionals unsure of how to effectively integrate play [5]. Although some of the participants came from hospitals employing trained staff (e.g. child life or health play specialists), they noted that these professionals were not available in every relevant clinical situation. To provide more healthcare professionals with skills in using play, consensus was reached that all healthcare professionals should have access to formal education on integrating play into paediatric practice. Our findings demonstrate that using play in clinical practice is engaged by all paediatric healthcare professions, rather than being limited to a few specialities. By expanding the description of why and how play can be integrated into paediatric hospitals, this study may support initiatives in generating goals for healthcare professionals’ training on how to use play in clinical practice. Such training can support healthcare professionals working in paediatric departments to provide care using a patient-centred approach to promote children’s rights in hospitals. To help break down barriers identified in this study, providing paediatric healthcare professionals with opportunities to learn more systematically about the value and benefits of play might be a fundamental future step (Table 4).

**Table 4:** Suggestions for future research priorities

• Explore the perspectives of paediatric patients and their families on the identified principles, competencies and learning objectives for play in hospitals
• Investigate opportunities for playful strategies in hospital settings for the adolescent group
• Evaluate the effect of interprofessional training of healthcare professionals in using playful strategies in paediatric clinical care

## Strengths and limitations

We used an electronic Delphi technique and translated the invitation letters and initial surveys into eight different languages, which allowed us to include the views of participants with limited English proficiency who are often excluded in international research. Although the participants’ views may not be representative of all healthcare professionals and practice settings, the inclusion of multiple nationalities and professions supported a broad range of opinions and perspectives on play in hospitals. A limitation of the Delphi panel was that it only represented high-income countries, which may limit the generalizability of our findings. However, these high-income countries are frontrunners in certain healthcare areas and may serve as inspiration for the innovative delivery of care. Three hospitals in the USA were invited to participate in our Delphi study but did not respond. The mean consensus threshold of ≥ 3 meant that consensus was reached regarding all statements in rounds 2 and 3. Using a higher threshold might not have yielded different results since only five statements had a mean rating < 4. One of the potential pitfalls of conducting multiple survey rounds is the probability of false consensus, which can arise due to fatigue, in which participants agree with statements just to end the process [12]. To mitigate the risk of false consensus, we predefined the number of survey rounds and informed participants before the study began. Another common bias in Delphi studies is the potential of low response rates and attrition between rounds [43]. By providing weekly reminders in the participants’ native language and conducting the survey rounds shortly after each other, we maintained a consistent response rate across all rounds (62–68%). A limitation is that the study’s design did not include the perspectives of families and patients, which should be included in future research.

## Conclusion

Using the Delphi method allowed us to identify common international principles and competencies for the use of play in paediatric clinical practice. We found that play can be used to communicate and build relationships with paediatric patients. Reaching consensus on principles and competencies for using play in hospitals is an important first step in developing a formal curriculum for an interprofessional education programme on how to use play in paediatric clinical practice. The insights from this study can help inform future patient-centred initiatives for healthcare professionals as play may be a way to communicate and involve paediatric patients appropriate for their age, stage of development and preferences for communication and engagement. It is essential to ensure that healthcare professionals can provide a patient-centred approach to protect the rights of paediatric patients in hospitals to potentially improve hospital experiences for paediatric patients and their families.

### Supplementary Information

Below is the link to the electronic supplementary material.

Supplementary file1 (PDF 237 KB)

Supplementary file2 (DOCX 183 KB)

## Data Availability

The data analyzed in this study is available from the corresponding author on reasonable request.

## Abbreviation

WHO: World Health Organization
