# Supplementary material for: Establishing consensus on principles and competencies for the use of play in clinical practice in hospitals: An international Delphi study
Source: Eur J Pediatr. 2024 Jan 6;183(4):1595–605. doi: 10.1007/s00431-023-05411-4 (PMC11001713; doi:10.1007/s00431-023-05411-4)
Supplement: Supplementary file 1 — Supplementary file1 (PDF 237 KB) [file 431_2023_5411_MOESM1_ESM.pdf]

Supplementary Table 1: First round questionnaire in English

Record ID

____

Please select the questionnaire in your language

- ◯ Deutsche
- ◯ English
- ◯ Español
- ◯ Nederlands
- ◯ Française
- ◯ Norsk
- ◯ Dansk
- ◯ Italiano

You have been appointed by your leader to contribute to a Delphi study aiming to better understand the use of play in hospitals. The first round of this Delphi study will gather opinions from international hospital staff on principles on the use of play in hospital.

These questions should take approximately 20-40 minutes. You may save and return to your answers before submitting them. By submitting the survey, you authorise the use of your anonymous responses to be collated at the group level. Your individual responses will not be shared with your leader, your organisation, or anyone outside of the designated research team.

We ask that you provide your honest, personal opinions about play. We encourage you to consider a broad, open-minded conceptualisation of play, and to call upon experiences and knowledge of playful activities, moments, opportunities, and situations.

Year of birth

____

Gender

- ◯ Male
- ◯ Female
- ◯ Non-Binary
- ◯ Other
- ◯ Do not wish to respond

What is the name of your hospital?

____

Please choose the profession that best describes your clinical background / training:

- ☐ Medical doctor
- ☐ Nurse
- ☐ Physiotherapist
- ☐ Occupational therapist
- ☐ Dietician / Nutritionist
- ☐ Psychologist
- ☐ Social worker
- ☐ Teacher
- ☐ Daycare teacher / Pedagogue
- ☐ Speech therapist
- ☐ Paediatric dentist
- ☐ Porter
- ☐ Radiographer
- ☐ Child life specialist / Play specialist
- ☐ Other

Please specify: ______________________

Are you a doctor undergoing specialist training?
- ◯ No
- ◯ Yes

Which medical specialty? ______________________

Have you fulfilled specialist training?
- ◯ No
- ◯ Yes

Which medical specialty? ______________________

Are you a specialised nurse?
- ◯ No
- ◯ Yes

Which medical specialty? ______________________

When did you complete your training (e.g. nurse, doctor, etc)

____
(Year)

When you think of "play in hospital", what comes to mind? Please feel free to include examples.

Do you regularly utilise play as part of your clinical work?
- ◯ No
- ◯ Yes

Do you consider play to be an integral part of delivering clinical care?
- ◯ No
- ◯ Yes

Did you learn to use play in your clinical work, either through training or by experience?	◯ No<br>◯ Yes, through training<br>◯ Yes, by experience<br>◯ Yes, both by training and experience

Please explain - where and/or by whom?

25%

Part 1

The rest of the questionnaire consists of questions according to four clinical contexts in which play is used in hospitals (based on this review). The four clinical contexts are shown in the figure below.

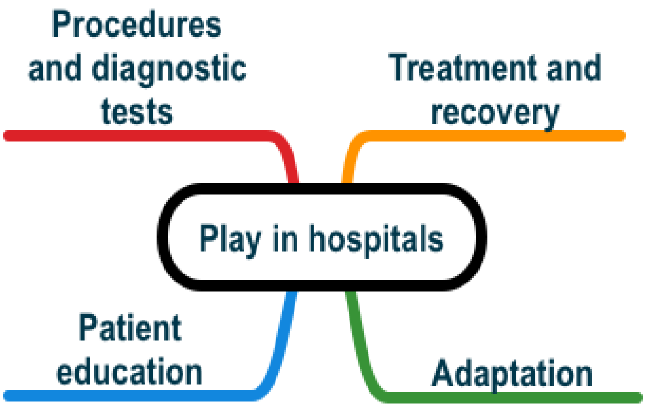

Adapted from: Gjærde, L.K., Hybschmann, J., Dybdal, D., Topperzer, M.K., Schrøder, M.A., Gibson, J.L., Ramchandani, P., Ginsberg, E.I., Ottesen, B., Frandsen, T.L., & Sørensen, J.L. (2021). Play interventions for paediatric patients in hospital: a scoping review. BMJ Open

We now start the first one.

For the following questions, please consider the use of play in procedures and diagnostic tests.

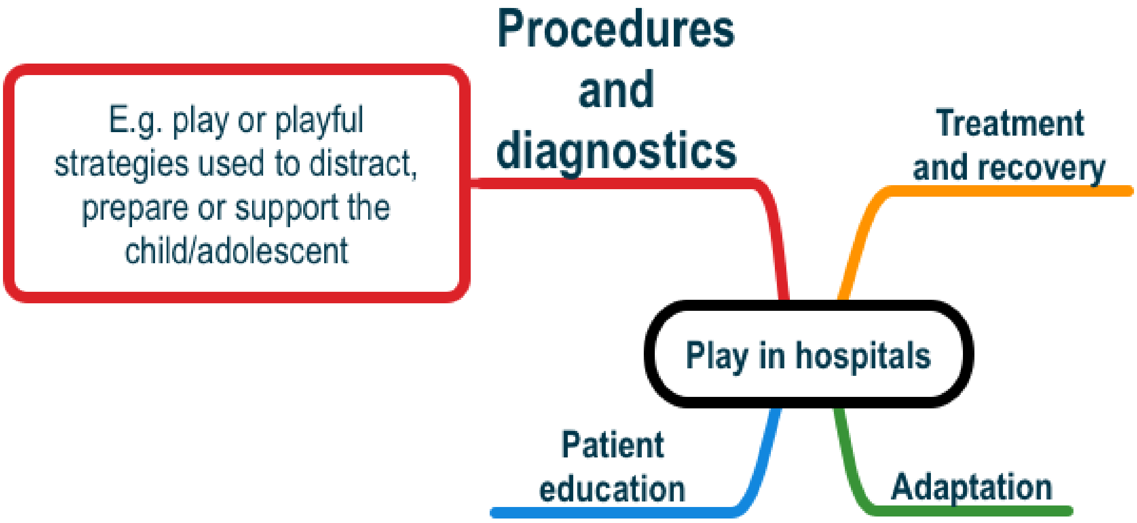

Do you use play during procedures or diagnostic tests?

◯ Procedures/diagnostic tests are not part of my clinical work
◯ Yes
◯ No

Why not? What are the barriers to your use of play during procedures or diagnostic tests?

How does play contribute to or support your procedural/diagnostic work?

Please give examples from your clinical practice of the use of play during procedures or diagnostic tests:

Which factors (e.g. age, previous experiences) do you take into account when using play in procedures and diagnostic tests?

Which factors facilitate your use of play during procedures or diagnostic tests?

What are the barriers to your use of play during procedures or diagnostic tests?

Part 2

We now turn to the second of the four clinical contexts in which play is used in hospitals.

For the following questions, please consider the use of play in Patient Education:

Use of play to increase knowledge, skills and attitudes (e.g. toward disease or treatment, the hospital environment, etc.)

40%

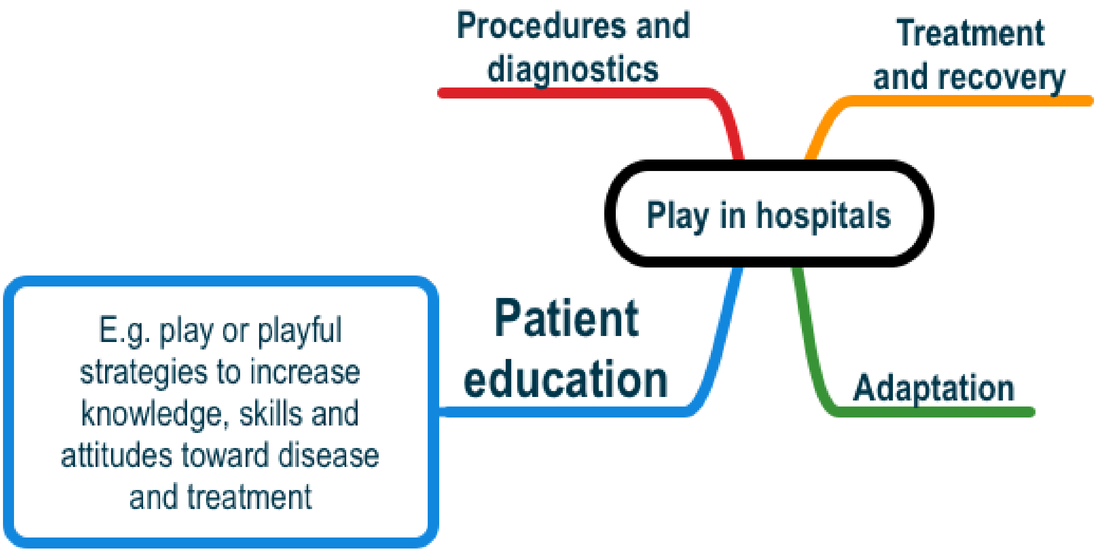

Do you use play in patient education?

◯ Patient education is not part of my clinical work
◯ Yes
◯ No

Why not? What are the barriers to your use of play in the context of patient education?

How does play contribute to or support your work in the context of patient education?

Please give examples from your clinical practice of the use of play in patient education:

Which factors (e.g. age, previous experiences) do you take into account when using play in the context of patient education?

Which factors facilitate your use of play in patient education?

What are the barriers to your use of play in the context of patient education?

55%

Part 3

We now turn to the third of the four clinical contexts in which play is used in hospitals.

For the following questions, please consider the use of play in Treatment and Recovery:

Considering play in the context of patient treatment or recovery/rehabilitation, either as a support or primary method

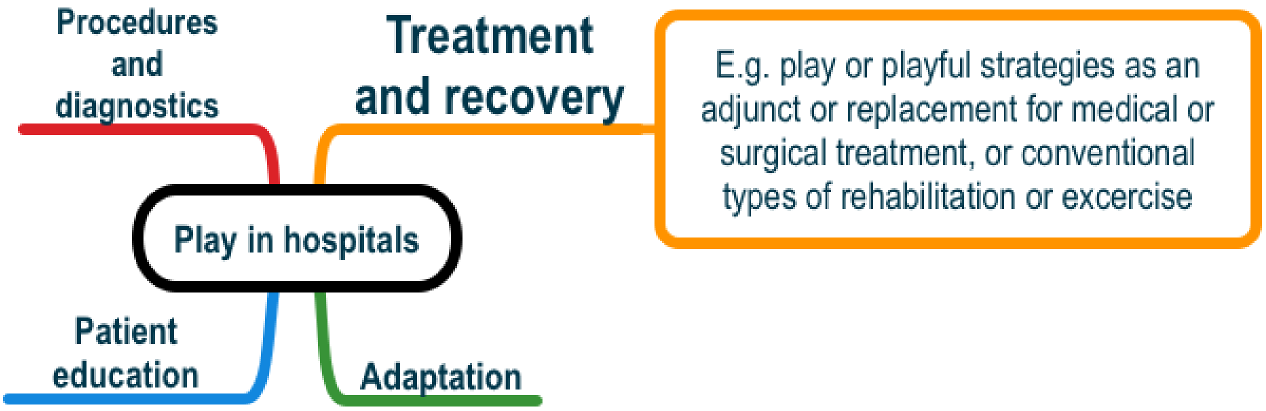

Do you use play in treatment and/or recovery?	◯ Treatment and/or recovery are not part of my clinical work<br>◯ Yes<br>◯ No

Why not? What are the barriers to your use of play in treatment and/or recovery?

How does play contribute to or support your work in the context of treatment and/or recovery?

Please give examples from your clinical practice of the use of play for treatment and/or recovery:

Which factors (e.g. age, previous experiences) do you take into account when using play in treatment and/or recovery?

Which factors facilitate your use of play in treatment and/or recovery?

What are the barriers to your use of play in treatment and/or recovery?

70%

Part 4

We now turn to the last of the four clinical contexts in which play is used in hospitals.

For the following questions, please consider the use of play in Adaptation (diversional or coping activities).

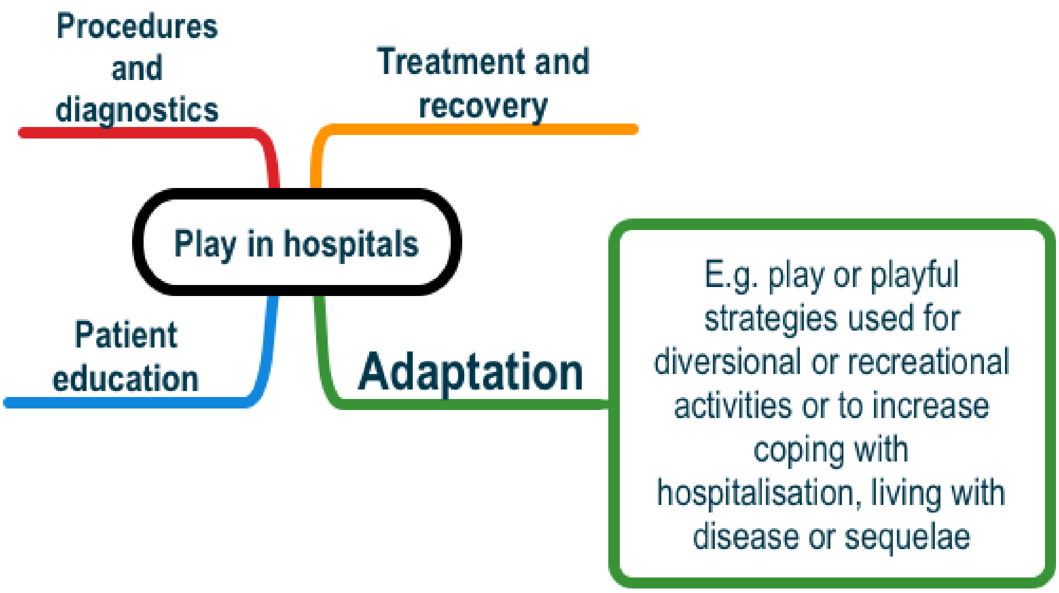

Do you use play in the context of diversional or coping activities?	◯ Diversional or coping activities are not part of my clinical work<br>◯ Yes<br>◯ No

Why not? What are the barriers to your use of play in the context of diversional or coping activities?

How does play contribute to or support your work in the context of diversional or coping activities?

Please give examples from your clinical practice of the use of play in the context of diversional or coping activities:

Which factors (e.g. age, previous experiences) do you take into account when using play in the context of diversional or coping activities?

Which factors facilitate your use of play in diversional or coping activities?

What are the barriers to your use of play in the context of diversional or coping activities?

85%

Final general questions about the use of play in clinical practice...

Are there circumstances in your clinical work where the use of play may be impractical or inappropriate? Please describe:

Are there any aspects of your clinical work that could benefit from play opportunities/engagement? Please describe:

Do you have any final thoughts to share on your experiences or perspectives on play in hospital?

100%
