# Supplementary material for: Establishing consensus on principles and competencies for the use of play in clinical practice in hospitals: An international Delphi study
Source: Eur J Pediatr. 2024 Jan 6;183(4):1595–605. doi: 10.1007/s00431-023-05411-4 (PMC11001713; doi:10.1007/s00431-023-05411-4)
Supplement: Supplementary file 2 — Supplementary file2 (DOCX 183 KB) [file 431_2023_5411_MOESM2_ESM.docx]

**Supplementary Figure 1. Screen shot of questionnaire design (Italian language example)**

**
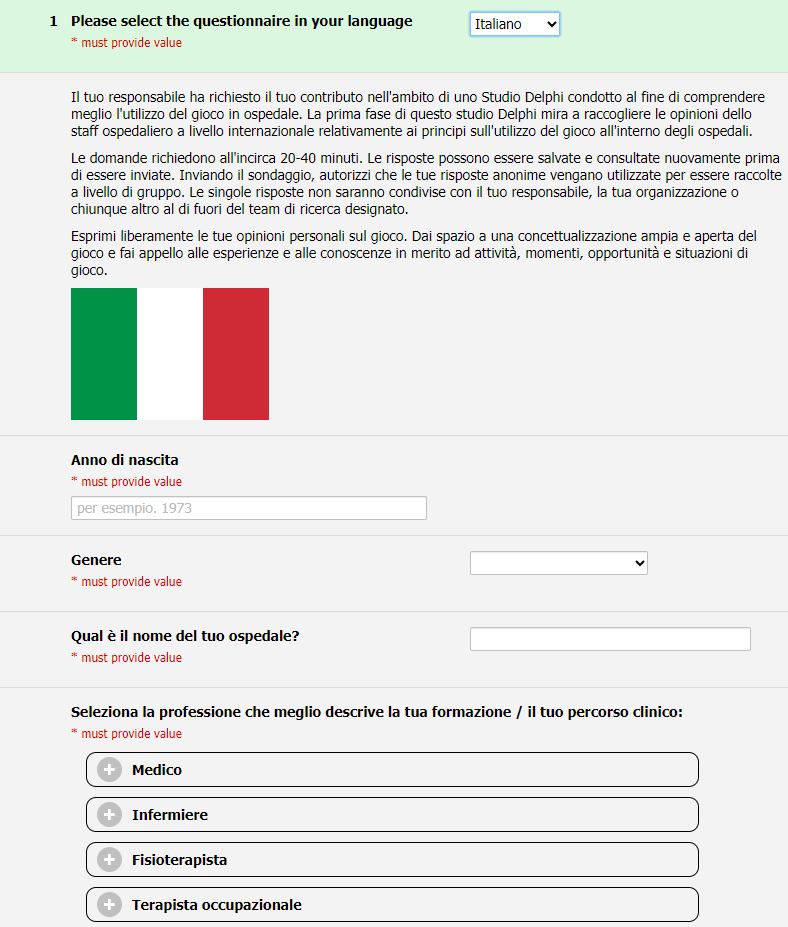
**

**Supplementary Figure 2. Flow diagram of the Delphi process**

Prepare invitation letter for **healthcare professionals** (participants) and 1^st^ survey round in English

Invitation letter for **hospital leaders** in English

Consensus

Recruitment phase

Research group ranked statements according to prominence

Round 3

Round 1

Round 2

Participants re-rated
Statements on a 5-points Likert Scale

Participants rated
English Statements on a
5-points Likert Scale

Preparational phase

Professional forward/backward translation to 8 languages

Leaders in specialised children’s hospital departments in 11 countries (Australia, Canada, Denmark, England, Germany, Ireland, Italy, Netherlands, Norway, UK, USA) receive an invitation letter (in English) and were asked to:

- Appoint 3-10 healthcare professionals to participate

*Inclusion criteria: knowledge on or using play in clinical work, healthcare professionals must have different professional backgrounds*

Appointed healthcare professionals (participants) receive invitation in Danish, Dutch, English, French, German, Norwegian, Italian, and Spanish

**Panel established**66 panelists

*45 of 66 responses )ændre)*

Participants responded to questionnaire with open-ended questions available in 8 different languages

Research group developed and formulated statements

*41 of 66 responses rat*

*41 of 66 responses*

Consensus
(defined as rating value ≥ 3)
on the concept of play in hospitals obtained

**Supplementary Table 2. Factors for using play in hospitals, ranked in order of importance**

| Factors | Round 2 | Round 3 |
| --- | --- | --- |
|  | N (%) | N (%) |
| 1. Mental/emotional condition of the patient | 35 (85) | 41 (100) |
| 2. Level of anxiety/distress in patient | 39 (95) | 39 (95) |
| 3. The patients age | 35 (85) | 39 (95) |
| 4. Physical condition of the patient | 28 (68) | 32 (78) |
| 5. Interests and hobbies of the patient | 28 (68) | 32 (78) |
| 6. Toys and activities available | 27 (66) | 29 (71) |
| 7. Family collaboration | 23 (56) | 23 (56) |
| 8. Patients previous hospital experiences | 22 (54) | 23 (56) |
| 9. Time available for preparing the play situation | 19 (46) | 24 (59) |
| 10. Room facilities | 14 (34) | 13 (32) |
| 11. Healthcare professional’s previous experiences | 12 (29) | 9 (22) |
| 12. Length of hospital admission | 9 (22) | 6 (15) |
| 13. Underlying illness trajectory | 18 (44) | 5 (12) |
| 14. Patients social background | 5 (12) | 5 (12) |
| 15. Team alignment regarding the use play | 7 (17) | 2 (5) |

**Supplementary Table 3. Barriers for not using play in hospitals, ranked in order of importance**

| Barriers | Round 2 | Round 3 |
| --- | --- | --- |
|  | N (%) | N (%) |
| 1. Time constraints | 30 (73) | 36 (88) |
| 2. Patients in critical health condition | 24 (59) | 34 (83) |
| 3. Lack of toys and activities available | 27 (66) | 32 (78) |
| 4. Staff shortage | 24 (59) | 28 (68) |
| 5. Lack of space in hospitals | 24 (59) | 26 (63) |
| 6. Lack of healthcare professionals experience in using play | 24 (59) | 25 (61) |
| 7. Unplanned appointments | 20 (49) | 20 (49) |
| 8. Lack of healthcare professional’s formal education in integrating play | 21 (51) | 18 (44) |
| 9. Patients with trauma/anxiety from previous experience | 13 (32) | 17 (42) |
| 10. Lack of creativity among healthcare professionals | 13 (32) | 13 (32) |
| 11. Lack of collaboration between healthcare professionals | 19 (46) | 12 (29) |
| 12. Limited communication between colleagues | 16 (39) | 8 (20) |
| 13. Parent skepticism towards the use of play | 16 (39) | 7 (17) |
| 14. Disagreement among colleagues regarding the value of play | 12 (29) | 7 (17) |
| 15. Patients attached to medical equipment/confined to bed | 12 (29) | 7 (17) |
| 16. Lack of collaboration between healthcare professionals and family members | 9 (22) | 5 (12) |

**Supplementary Table 4. Facilitators for using play in hospitals, ranked in order of importance**

| Facilitators | Round 2 | Round 3 |
| --- | --- | --- |
|  | N (%) | N (%) |
| 1. Knowledge about the child (interest, hobbies, social background etc.) | 25 (61) | 37 (90) |
| 2. Trustful relationships with the patients | 25 (61) | 36 (88) |
| 3. Access to appropriate toys and activities | 31 (76) | 31 (76) |
| 4. Sufficient time | 18 (44) | 31 (76) |
| 5. Collaboration with parents | 21 (51) | 27 (66) |
| 6. Hospital settings encouraging play | 20 (49) | 23 (56) |
| 7. Existing play culture in department | 18 (44) | 19 (46) |
| 8. Trustful relationships with the families | 16 (39) | 19 (46) |
| 9. Healthcare professionals’ experiences in using play | 15 (37) | 15 (37) |
| 10. Adequate preparation time | 13 (32) | 11 (27) |
| 11. Team agreement of the importance of play | 12 (29) | 11 (27) |
| 12. Feeling empathy for the child and the family | 11 (27) | 10 (24) |
| 13. Cleary defined roles between staff members regarding play | 9 (22) | 8 (20) |
| 14. Healthcare professionals with good clinical skills | 11 (27) | 7 (17) |
| 15. Interdisciplinary collaboration | 9 (22) | 7 (17) |
| 16. Patients initiating play or activities themselves | 8 (20) | 7 (17) |
| 17. Family engagement | 12 (29) | 6 (15) |
| 18. A calm and quiet department | 10 (24) | 6 (15) |
| 19. Acknowledgement of the use of play by hospital management | 11 (27) | 5 (12) |
| 20. Patients bringing their own toys or activities | 4 (10) | 3 (7) |
| 21. Exchange of experiences in using play between staff members | 9 (22) | 2 (5) |
| 22. Adequate communication between colleagues | 6 (15) | 2 (5) |
| 23. Previous interactions and experiences with the pediatric patient | 10 (24) | 1 (2) |
